# Metropolitan Consumption Hospitals and the Insurance Act

**Published:** 1913-03-01

**Authors:** 


### METROPOLITAN CONSUMPTION HOSPITALS AND THE INSURANCE ACT.

One of last Saturday's daily papers contained news throwing much light upon how "sanatorium benefit" under the Insurance Act is affecting Metropolitan consumption hospitals. On an outside page appeared a large advertisement that the well-known institution at Brompton was in debt to the tune of £10,000; elsewhere was half a column to the effect that the Committee of Mount Vernon Hospital, alarmed at the shrinkage of their subscription list, have been obliged to suspend all gratuitous treatment and have further almost decided to sell their headquarters at Hampstead, continuing only with the work at Northwood. Already at the latter place only such cases are admitted as can pay twenty-five shillings per week. Facts such as these naturally suggest inquiry as to whether the institution in question has had any custom, so to say, from the London and other Insurance Committees; and it appears that it has, since all but forty beds at Hampstead have been given up to insurance patients. If, then, in spite of this, the closing down and disposal of that building is contemplated, the only conclusion can be that the fees paid for these cases are inadequate; either relatively, owing to high cost of maintenance, or actually at the figures mentioned under the Metropolitan Asylums Board scheme.

Here are the two chief consumption hospitals in London, justly proud of their standing, prestige, and experience; ante-dating the two great recent developments in tuberculosis work—namely, the sanatorium and tuberculin; modelled, therefore, on the old style, with massive buildings, troops of nurses, chapels, much regard for appearance, and so forth; managed by not very democratic boards. Upon this scene enter very suddenly the representatives of a new state of affairs, a new regime backed (be it well noted), not only by the law of the land and the resources of the State, but also by very practical authorities like Mr. Garland and Sir R. W. Philip, people who not only say but realise in everything they do that tuberculosis is of all things an economic problem, a matter of making pounds, shillings, and pence go as far as ever they will; people who have got beyond giving bottles of medicine in an out-patient department and governors' letters admitting just a few to the stately wards of an eighty thousand pound edifice; who are grappling with the tasks of preventing home infection, of looking after dependants, of providing employment, and other practical and pressing affairs which are not so much corollaries as pre-requisites, if consumption in the working classes is to be touched at all.

It is obviously a vast convenience to have a great established body like the Metropolitan Asylums Board to take over the care of the consumptives of London; but what as yet are their experience, qualifications, and accommodation for such a task? To take one detail, we see that, as we had anticipated, they have appointed one of their own junior medical officers to the resident charge of the Downs Schools at Sutton, which have been converted into a sanatorium. This gentleman, although no doubt entirely worthy of promotion, seems not to have had any previous special experience of tuberculosis. He will, of course, be familiar with the reputedly rather meticulous administrative methods of the authority he serves; but here is a source of danger to these particular newcomers just as great as an independent attitude must be to those previously in exclusive possession of the field of labour. You cannot by Act of Parliament change a law of Nature, it has been said; and what holds good in the natural holds good equally in the pathological sphere. The methods found to suit the care of children acutely ill with diphtheria cannot be the same as those best for moderately consumptive adults. Nor do we very greatly value the safeguard of supervision by a visiting physician. All the famous sanatoriums have made their name through devoted personal service of a resident medical man —be it Bodington or Brehmer or Trudeau or Walther or the first medical superintendent at Frimley. Dr. Latham and Mr. Garland (neither of them with an axe to grind) sum this up fairly emphatically in their book, "The Conquest of Consumption": "Anyone who has a real knowledge of the requirements of sanatorium treatment recognises that the whole success of the results obtained depends upon the capacity and character of the medical man in charge."

Meanwhile, it is a great pity even to hear talk of allowing well-found (rather too well-found, perhaps) institutions to go down. All that can be done is to urge conciliation and compromise, compromise and conciliation, again and again. Any measure of the far-reaching nature of the National Insurance Act, even if medical opinion had been properly consulted, and if the Bill had not been "rushed through," would have been bound to result in awkward situations such as that above noticed. But whatever happens, the larger question of the formally unrecognised importance of the medical institution must gain in prominence.

